# Supplementary material for: Network-based diffusion analysis reveals context-specific dominance of dance communication in foraging honeybees
Source: Nat Commun. 2020 Jan 31;11:625. doi: 10.1038/s41467-020-14410-0 (PMC6994492; doi:10.1038/s41467-020-14410-0)
Supplement: Supplementary file 1 — Supplementary Information [file 41467_2020_14410_MOESM1_ESM.pdf]

Supplementary Information

Network-based diffusion analysis reveals context-specific dominance of dance communication in foraging honeybees

Hasenjager et al.

Table of Contents:

Supplementary Table 1 (p. 2)
Supplementary Table 2 (p. 2)
Supplementary Table 3 (p. 3)
Supplementary Table 4 (p. 3)
Supplementary Table 5 (p. 4)
Supplementary Table 6 (p. 4)
Supplementary Figure 1 (p. 5)
Supplementary Table 7 (p. 6)
Supplementary Table 8 (p. 7)
Supplementary Table 9 (p. 8)
Supplementary Note 1 (pp. 9-10)
Supplementary Table 10 (p. 11)
Supplementary Table 11 (p. 11)
Supplementary Table 12 (p. 12)
Supplementary Table 13 (p. 13)
Supplementary Note 2 (pp. 14-15)
Supplementary References (p. 15)

Supplementary Table 1. Support (Σw_i) for weighting the dance-following network in the Recruitment NBDA by either the number of waggle runs that were followed or by the total duration of time spent following dancers.

Dance-following network	Σw_i
Number of waggle runs followed	0.872
Duration of time following dances (sec)	0.128

The model set from which these values were obtained was assembled following the same procedure as described in the Methods (main text), with the addition of a set of models that included the alternate dance-following network.

Supplementary Table 2. Model set evaluating the relative support for interactions between different network types influencing recruitment order to the FULL feeder.

s parameters	-log(*L*)	*K*	AICc	ΔAICc
s_{Dance}	184.20	2	372.63	0
$s_{\text{Dance}} + s_{\text{Trophallaxis}}$	184.20	3	374.87	2.24
$s_{\text{Dance}} + s_{\text{Trophallaxis}} + s_{\text{Dance*Trophallaxis}}$	184.20	4	377.19	4.56
$s_{\text{Dance}} + s_{\text{Antennal Contact}}$	184.20	3	374.87	2.24
$s_{\text{Dance}} + s_{\text{Antennal Contact}} + s_{\text{Dance*Antennal Contact}}$	183.21	4	375.20	2.57

Due to the strong support it received (Supplementary Table 7), all models included an effect of the number of visits to the EMPTY feeder during the trial on the rate of social transmission. For each model, the negative log-likelihood, number of estimated parameters (*K*), AICc, and the difference in AICc relative to the top-ranked model (ΔAICc) are provided. Although ΔAICc values of ~2 generally indicate some support for a model, the similarity in -log(*L*) across all models indicates that increased model complexity did not improve model fit; rather, ΔAICc values were close simply due to the addition of an extra parameter[1].

Supplementary Table 3. Dance-following bouts by marked individuals for natural food sources and for the FULL feeder during the first 20 min of each trial.

Colony	Trial date	Dances followed for natural sources	Dances followed for the FULL feeder
1	Aug. 29th	63	27
2	Sept. 6th	1	47
3	Sept. 23rd	132	1
4	Oct. 5th	123	3

The marked increase in dancing for natural food sources in colonies 3 and 4 coincided with the typical emergence time of ivy (*Hedera* spp.) in southern England[2,3].

Supplementary Table 4. Support (Σw_i) for social transmission pathways in the Recruitment NBDA using data from only the first two trials.

Social network[a]	Σw_i
Dance-following network	> 0.999
Trophallaxis network (duration)	0.289
Trophallaxis network (number of interactions)	0.289
Antennal contact network (duration)	0.289
Antennal contact network (number of interactions)	0.289
Homogeneous network[b]	< 0.001
No network (asocial learning only)[c]	< 0.001

a: Variants were considered for both the trophallaxis and antennal contact networks in which network connections were weighted either by total interaction duration (sec) or according to the number of separate interactions (regardless of their duration) between two individuals.
b: The homogeneous network included a connection with a strength of 1 between every forager upon its first return to the hive from the FULL feeder and all potential recruits that had not yet departed the hive for the FULL feeder. Support for this network would have indicated that none of the measured social networks provided a sufficient approximation for the true pathway(s) of social transmission[4].
c: Asocial models assume that feeder discovery occurred through independent search alone, without relying on social information. Note that asocial learning is also assumed to operate within models that include a social transmission component[5].

Supplementary Table 5. Estimated rates of social transmission (*s*) in the Recruitment NBDA using data from only the first two trials.

Social transmission parameter, *s*	Model-averaged estimate (95% CI)[a]
Dance-following *s*	3.6 (0.49, 71.50)
Trophallaxis (number of interactions) *s*	0 (0, 0.38)
Antennal contact (duration) *s*	0 (0, 0.41)

a: Model-averaged estimates were obtained using the variants specified above for the trophallaxis and antennal contact networks due to these variants receiving more support (Σw_i) than their alternatives; see Methods for further details. Confidence intervals were obtained using profile likelihood techniques[6] from the highest ranked model that included a given network. These values correspond to an estimated 96.5% (95% CI: 89.1%, 97.9%) of recruitment events (*n* = 49) resulting from dance-following interactions, < 0.01% (95% CI: 0%, 1.8%) from trophallaxis, and < 0.01% (95% CI: 0%, 42.3%) from antennal contact.

Supplementary Table 6. Overall support (Σw_i), model-averaged estimates (MAE), and 95% CIs for additional predictor variables included in the Recruitment NBDA using data from only the first two trials.

Predictor variable	Σw_i	MAE (95% CI)
Trial 2 (asocial effect)	0.382	0 (-)
Trial 2 (social effect)	0.306	0.09 (-0.47, 0.91)
Number of visits to EMPTY feeder (asocial effect)	0.261	-0.18 (-2.1, 1.28)
Number of visits to EMPTY feeder (social effect)	0.755	-0.16 (-0.40, -0.03)

Predictor variables could influence recruitment by modifying either the baseline rate of feeder discovery through independent search (asocial effect) or the rate of social transmission (social effect). The baseline discovery rate was set to that of an individual in trial 1 that returned to the EMPTY feeder an average number of times during the trial. See Supplementary Table 7 for explanation regarding interpretation of effects. Confidence intervals were obtained using profile likelihood techniques[6] conditional on the best supported model that included a given parameter. It was not possible to obtain 95% CIs for estimates of trial-specific asocial learning rates, as any model with reasonable support ($w_i > 0.01$) that included these effects failed to converge.

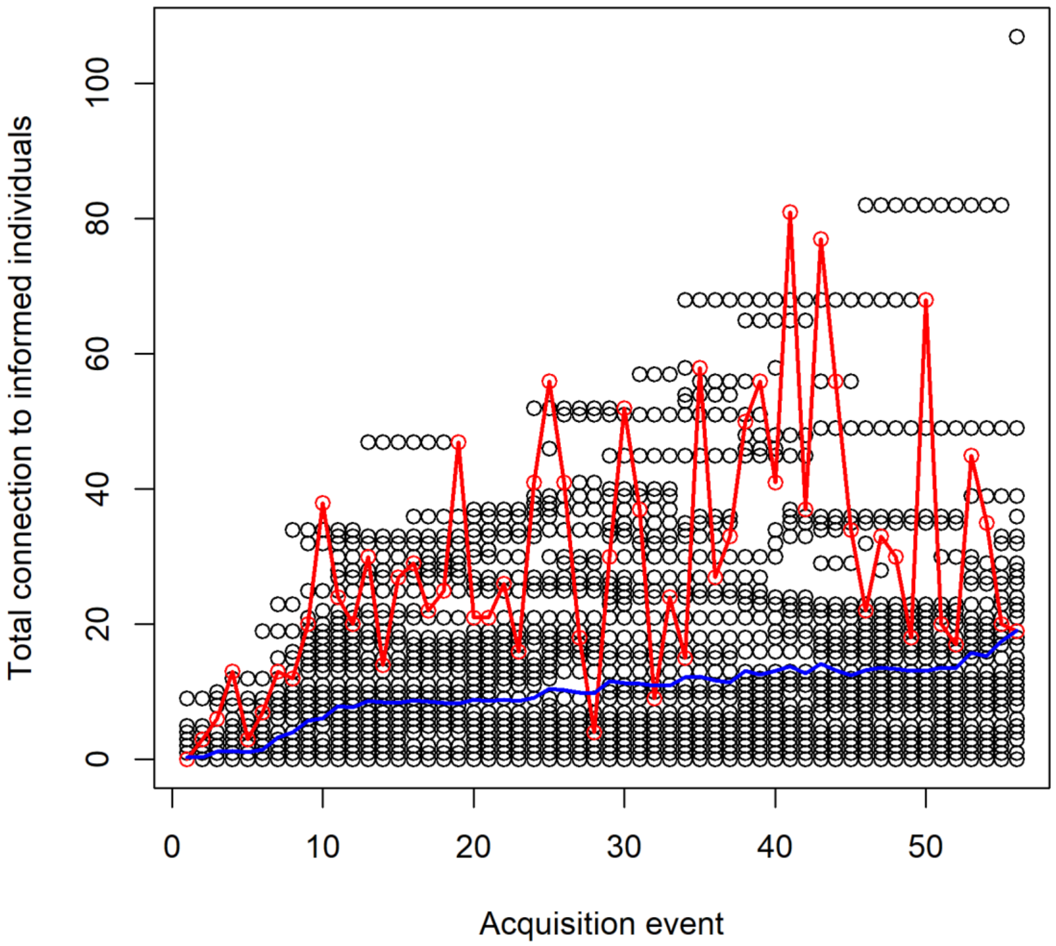

Supplementary Figure 1. The order in which recruits discovered the FULL feeder was predicted by the number of waggle runs that they followed for it. The total connection of naïve foragers to informed foragers—i.e., the total number of waggle runs a potential recruit followed for the feeder from the start of the trial up to each feeder discovery event—is plotted against the sequence of discovery events. The individual that discovered the feeder at each event is plotted in red. In the absence of social transmission, the path of the red line would be expected to match that of the blue line, which indicates the average connection to informed foragers. That the red line is above the blue line for nearly all feeder discovery events indicates that social transmission followed the waggle dance network. Source data are provided as a Source Data file.

Supplementary Table 7. Overall support (Σw_i), model-averaged estimates (MAE), and 95% CIs for additional predictor variables included in the Recruitment NBDA.

Predictor variable	Σw_i	MAE (95% CI)
Trial ID		
Trial 2 (asocial effect)	0.119	0 (-)
Trial 2 (social effect)	0.171	0.03 (-0.51, 0.85)
Trial 3 (asocial effect)	0.119	0 (-)
Trial 3 (social effect)	0.171	0.03 (-1.22, 1.21)
Trial 4 (asocial effect)	0.119	0 (-)
Trial 4 (social effect)	0.171	-0.18 (-2.58, 0.22)
Number of visits to EMPTY feeder (asocial effect)	0.25	-0.12 (-2.37, 1.23)
Number of visits to EMPTY feeder (social effect)	0.947	-0.25 (-0.44, -0.08)

Predictor variables could influence recruitment by modifying either the baseline rate of feeder discovery through independent search (asocial effect) or the rate of social transmission (social effect). The baseline discovery rate was set to that of an individual in trial 1 that returned to inspect the EMPTY feeder an average number of times during the trial. The above estimates provide the linear effect of a given parameter on the log scale; thus, a potential recruit that visited the EMPTY feeder four times would experience a social transmission rate exp (3 * -0.25) = 0.47 times less than that of an individual that had only visited it once. Confidence intervals were obtained using profile likelihood techniques conditional on the best supported model that included a given parameter[6]. It was not possible to obtain 95% CIs for estimates of trial-specific asocial learning rates, as any model with reasonable support ($w_i > 0.01$) that included these effects failed to converge.

Supplementary Table 8. Model set evaluating the relative support for interactions between different network types influencing reactivation order to the FULL feeder.

s parameters	-log(L)	K	AICc	ΔAICc
$s_{\text{Dance}} + s_{(\text{Trophallaxis + Antennal Contact})}$	179.49	9	380.13	0
$s_{\text{Dance}} + s_{(\text{Trophallaxis + Antennal Contact})} + s_{\text{Trophallaxis*Dance}}$	179.24	10	382.41	2.28
$s_{\text{Dance}} + s_{(\text{Trophallaxis + Antennal Contact})} + s_{\text{Antennal Contact*Dance}}$	179.25	10	382.43	2.30
$s_{\text{Dance}} + s_{(\text{Trophallaxis + Antennal Contact})} + s_{\text{Trophallaxis*Antennal Contact}}$	179.46	10	382.86	2.73

Due to the strong support they received (Supplementary Table 9), all models included the following effects: trial ID (asocial effect), trial ID (social effect), and feeder experience (asocial effect). $s_{(\text{Network 1 + Network 2})}$ means that the rates of social transmission for these networks were constrained to be equal. For each model, the negative log-likelihood, number of estimated parameters (K), AICc, and the difference in AICc relative to the top-ranked model (ΔAICc) are provided. Although ΔAICc values of ~2 generally indicate some support for a model, the similarity in -log(L) across all models indicates that increased model complexity did not improve model fit; rather, ΔAICc values were close simply due to the addition of an extra parameter[1].

Supplementary Table 9. Overall support (Σw_i), model-averaged estimates (MAE), and 95% CIs for additional predictor variables included in the FULL feeder Reactivation NBDA.

Predictor variable	Σw_i	MAE (95% CI)
Trial ID		
Trial 2 (asocial effect)	0.994	1.53 (0.07, 3.94)
Trial 2 (social effect)	0.957	-2.73 (-4.96, -1.32)
Trial 3 (asocial effect)	0.994	-23.96 (-∞, -1.42)
Trial 3 (social effect)	0.957	0.01 (-1.32, 2.06)
Trial 4 (asocial effect)	0.994	-0.81 (-3.99, 1.51)
Trial 4 (social effect)	0.957	-0.14 (-1.22, 2.03)
Days of feeder experience (asocial effect)	0.923	1.22 (0.51, 2.59)
Days of feeder experience (social effect)	0.5	0.21 (-0.12, 0.74)

Predictor variables could influence the rate of reactivation by modifying either the baseline rate of reactivation (asocial effect) or the effect of social interactions over reactivation (social effect). The baseline reactivation rate was set to that of a forager from Trial 1 with the average number of days of experience at the FULL feeder. See Supplementary Table 7 for explanation regarding interpretation of effects. Confidence intervals were obtained using profile likelihood techniques[6] conditional on the best supported model that included a given parameter.

Supplementary Note 1. Reactivation of trained foragers to the EMPTY feeder

In addition to examining the reactivation of foragers to the FULL feeder, our experimental setup allowed us to also investigate reactivation in one further context: reactivation to the EMPTY feeder. As both feeders had provided identically scented sucrose on the day immediately preceding the trial, collection of sucrose containing that scent from one site (the FULL feeder) may have elicited reactivation of foragers that had experienced that scent at the EMPTY feeder[7]. In addition, waggle dances have been shown to reactivate bees not only to the indicated site, but to any site that the follower has previously visited[8,9].

Following the protocols described in the main text, we constructed interaction networks that quantified dance-following interactions, trophallaxis, and antennation events between foragers returning from the FULL feeder and foragers that had been trained to the EMPTY feeder. While individuals that reactivated to the EMPTY feeder became informed (in the sense that they acquired current knowledge regarding their familiar feeder's profitability), we assumed they did not begin transmitting information that might serve to reactivate others until they were recruited to the FULL feeder. This is because individuals returning from the EMPTY feeder did not produce waggle dances, nor did they return with scented sucrose solution in their crop.

Across all 4 trials, 89 individuals were reactivated to the EMPTY feeder. However, we restricted the NBDA to a subset of 70 reactivation events. We excluded as potential learners 18 individuals that were present at the EMPTY feeder when it was put in place (or shortly thereafter) or were not observed in the hive prior to arriving at the EMPTY feeder. This was done so that our analysis was restricted to the subset of individuals that could have engaged in our focal interaction types (regardless of whether they in fact did so). We also excluded an individual that, after reactivating to the EMPTY feeder, appeared to fly directly to the FULL feeder without first returning to the hive (having apparently acquired detailed enough spatial information for the FULL feeder's location prior to reactivation). As successful recruitment typically requires longer and/or more frequent periods of interaction with successful foragers than does reactivation[10,11], this individual had especially strong network connections relative to the rest of their cohort. For similar reasons, five individuals that were recruited to the FULL feeder without having first been reactivated to the EMPTY feeder were not considered as potential learners in this analysis. However, note that inclusion of these 6 individuals as potential learners does not qualitatively alter our findings.

The NBDA provided unequivocal evidence indicating that patterns of antennation—specifically the number of antennation events ($\Sigma w_i = 0.999$; Supplementary Table 10)—predicted the order in which individuals were reactivated to the EMPTY feeder. The trophallaxis network also received some support when connections were weighted by the number of trophallactic events ($\Sigma w_i = 0.639$), rather than their duration ($\Sigma w_i = 0.272$; Supplementary Table 10). However, in contrast to the findings for the FULL feeder, the dance network received very little support ($\Sigma w_i = 0.233$; Supplementary Table 10). Models of homogeneous social transmission ($\Sigma w_i < 0.001$) and those that treated reactivation as a purely asocial process ($\Sigma w_i < 0.001$) both received virtually no support (Supplementary Table 10).

Model-averaged estimates and 95% CIs for the social transmission parameters are presented in Supplementary Table 11. Of the 70 reactivation events considered here, 72% (95% CI: 52.7%, 88.5%) were explained by patterns of antennal contact and 16.5% (95% CI: 5.7%, 33.9%) by trophallaxis. At best, only 4.4% were explained by patterns of dance-following (<

0.01%; 95% CI: 0%, 4.4%). An estimated 11.6% of these reactivations occurred independently of the transmission pathways considered here. Thus, foragers collecting from one site successfully reactivated the majority of individuals to a different, spatially distant site. This outcome most likely resulted from individuals detecting sucrose and/or food-associated odours borne on foragers' bodies through antennation (and trophallaxis to a lesser extent).

Dances for the FULL feeder appeared to be ineffective in eliciting reactivation to the EMPTY feeder, suggesting that followers may have detected a mismatch between their familiar foraging site and the spatial information conveyed by the dance. Conversely, dance-following interactions predicted reactivation patterns for individuals that experienced no such mismatch (i.e., those trained to the FULL feeder; Table 4).

Although we found evidence for trial-specific social transmission rates (social effect: $\Sigma w_i > 0.999$; Supplementary Table 12), the amount of experience at the EMPTY feeder did not influence the order of reactivation (asocial effect: $\Sigma w_i = 0.237$; social effect: $\Sigma w_i = 0.247$; Supplementary Table 12). We also did not find evidence of interactive effects between these networks influencing the order of reactivation (Supplementary Table 13).

Supplementary Table 10. Support (Σw_i) for social transmission pathways in the EMPTY feeder Reactivation NBDA.

Social network[a]	Σw_i
Dance-following network	0.233
Trophallaxis network (duration)	0.272
Trophallaxis network (number of interactions)	0.639
Antennal contact network (duration)	0.001
Antennal contact network (number of interactions)	0.999
Homogeneous network[b]	< 0.001
No network (asocial learning only)[c]	< 0.001

a: Variants were considered for both the trophallaxis and antennal contact networks in which network connections were weighted either by total interaction duration (sec) or according to the number of separate interactions (regardless of their duration) between two individuals.
b: The homogeneous network included a connection with a strength of 1 between every forager upon its first return to the hive from the FULL feeder and all individuals that had yet to reactivate to the EMPTY feeder and had not yet departed the hive for it. Support for this network would have indicated that none of the measured social networks provided a sufficient approximation for the true pathway(s) of social transmission[4].
c: Asocial models assume that reactivation was entirely self-initiated, without relying on social information. Note that asocial reactivation is also assumed to operate within models that include a social transmission component[5].

Supplementary Table 11. Estimated rates of social transmission (*s*) in the EMPTY feeder Reactivation NBDA.

Social transmission parameter, *s*	Model-averaged estimate (95% CI)[a]
Dance-following *s*	0.001 (0, 12.98)
Trophallaxis (number of interactions) *s*	11.89 (3.24, 40.21)
Antennal contact (number of interactions) *s*	9.11 (3.24, 40.21)

a: Model-averaged estimates were obtained using the variants specified above for the trophallaxis and antennal contact networks due to these variants receiving more support (Σw_i) than their alternatives; see Methods for further details. Confidence intervals were obtained using profile likelihood techniques[6] from the highest ranked model that included a given network. The highest ranked model included both $s_{\text{trophallaxis}}$ and $s_{\text{antennal contact}}$ and constrained their estimates to be equal.

Supplementary Table 12. Overall support (Σw_i), model-averaged estimates (MAE), and 95% CIs for additional predictor variables included in the EMPTY feeder Reactivation NBDA.

Predictor variable	Σw_i	MAE (95% CI)
Trial ID		
Trial 2 (asocial effect)	0.037	0 (-8.9 x 10^{-5}, 0.0001)
Trial 2 (social effect)	> 0.999	-0.63 (-0.89, -0.36)
Trial 3 (asocial effect)	0.037	0 (-0.0005, 0.0003)
Trial 3 (social effect)	> 0.999	-1.93 (-2.52, -1.33)
Trial 4 (asocial effect)	0.037	0 (-0.0005, 0.0003)
Trial 4 (social effect)	> 0.999	-2.27 (-2.9, -1.63)
Days of feeder experience (asocial effect)	0.237	-0.02 (-0.09, 0.05)
Days of feeder experience (social effect)	0.247	0.01 (-0.001, 0.01)

Predictor variables could influence the rate of reactivation by modifying either the baseline rate of reactivation (asocial effect) or the effect of social interactions over reactivation (social effect). The baseline reactivation rate was set to that of a forager from Trial 1 with the average number of days of experience at the EMPTY feeder. See Supplementary Table 7 for explanation regarding interpretation of effects. As only a small number of models with low Akaike weights failed to produce standard errors for these parameters, unconditional standard errors were able to be obtained. These were then used to calculate Wald confidence intervals adjusted to account for model selection uncertainty[1].

Supplementary Table 13. Model set evaluating the relative support for interactions between different network types influencing reactivation order to the EMPTY feeder.

s parameters	-log(*L*)	*K*	AICc	ΔAICc
$s_{(\text{Trophallaxis + Antennal Contact})}$	193.22	4	395.06	0
$s_{(\text{Trophallaxis + Antennal Contact})} + s_{\text{Trophallaxis*Antennal Contact}}$	193.1	5	397.14	2.09
$s_{(\text{Trophallaxis + Antennal Contact})} + s_{\text{Dance}}$	193.22	5	397.38	2.32
$s_{(\text{Trophallaxis + Antennal Contact})} + s_{\text{Dance}} + s_{\text{Dance*Trophallaxis}}$	192.49	6	398.32	3.27
$s_{(\text{Trophallaxis + Antennal Contact})} + s_{\text{Dance}} + s_{\text{Dance*Antennal Contact}}$	193.22	6	399.77	4.72

Due to the strong support they received (Supplementary Table 12), all models included trial-specific social transmission rates. $s_{(\text{Network 1 + Network 2})}$ means that the rates of social transmission for these networks were constrained to be equal. For each model, the negative log-likelihood, number of estimated parameters (*K*), AICc, and the difference in AICc relative to the top-ranked model (ΔAICc) are provided. Although ΔAICc values of ~2 generally indicate some support for a model, the similarity in -log(*L*) across all models indicates that increased model complexity did not improve model fit; rather, ΔAICc values were close simply due to the addition of an extra parameter[1].

Supplementary Note 2. The NBDA Model

The basic NBDA model states that an individual *i* at time *t* acquires a target behavioural pattern at rate:

$$\lambda_i(t) = \lambda_0(t)\big(1 - z_i(t)\big)\left(s\sum_{j=1}^{N} a_{ij}z_j(t) + 1\right) \tag{1}$$

where $\lambda_0(t)$ is the baseline rate of acquisition, $z_i(t)$ is the status of *i* at time *t* (1 = informed; 0 = naïve), s is the rate of social transmission, a_{ij} is network connection from *j* to *i*, and *N* is the number of individuals in the network. The model assumes that an individual acquires the behaviour through social transmission at a rate proportional to its connection to informed individuals, $\sum_{j=1}^{N} a_{ij}z_j(t)$. Thus, s estimates the rate of social transmission per unit of network connection with informed individuals, relative to the baseline acquisition rate. The $z_j(t)$ and $z_i(t)$ terms respectively ensure that only informed individuals can transmit information and only naïve individuals can learn.

Here, we expand this model to allow for the incorporation of multiple, time-varying networks, as well as other predictor variables that may influence asocial and/or social learning:

$$\lambda_i(t) = \lambda_0(t)\big(1 - z_i(t)\big)\left(\exp\big(\Gamma_i(t)\big)\sum_{n} s_n \sum_{j=1}^{N} a_{n,ij}(t)z_j(t) + \exp\big(B_i(t)\big)\right) \tag{2}$$

$$\mathrm{B}_i(t) = \sum_{k=1}^{V} \beta_k x_{k,i}(t) \tag{3}$$

$$\Gamma_i(t) = \sum_{k=1}^{V} \gamma_k x_{k,i}(t) \tag{4}$$

where s_n estimates the rate of social transmission through network *n*, $a_{n,ij}(t)$ is the network connection between *j* and *i* in network *n* at time *t*, $x_{k,i}(t)$ is the value for the kth variable for individual *i* at time *t*, β_k is the effect of variable *k* on asocial learning, and γ_k is the effect of variable *k* on social transmission. The impact of variable *k* on learning rates is modelled as a linear effect on the log scale, as is common in survival analysis. Previous versions of NBDA allowed predictor variables to either influence only the asocial learning rate (an 'additive' model) or influence asocial and social learning rates in the same way (a 'multiplicative' model)[5]. The above formulation allows for models in which each predictor can either influence asocial learning only ($\gamma_k = 0$), influence social transmission only ($\beta_k = 0$), or influence both asocial and social learning to differing degrees ($\beta_k \neq \gamma_k$).

Note that when a dynamic observation/interaction network is used (as in the current study) $\Sigma_{j=1}^{N} a_{n,ij}(t)$ can be reduced down to $\Sigma_{j=1}^{N} o_{n,i}(t)$, where *o* is the number of times that individual *i* engaged in interaction type *n* (e.g., the number of waggle runs followed for the FULL feeder), regardless of the identity of the demonstrator. Thus, it is possible to describe this model without the use of a network. Nevertheless, there are several advantages to building this model within the NBDA framework. The first is practical, as the NBDA package is already set up to analyze such data. Simply including $\Sigma_{j=1}^{N} o_{n,i}$ as a predictor

variable in a standard survival analysis package (e.g., using the Cox model) would change the relationship between social interactions and learning rate—i.e., the learning rate would be proportional to $\exp(\Sigma_{j=1}^{N} o_{n,i})$ rather than to $\Sigma_{j=1}^{N} o_{n,i}$—meaning that researchers would be required to write custom functions[5]. Second, retaining interaction data in network form allows for more easily extending the analysis to test for differences in social transmission by breaking down the network into different learning pathways. For example, a network of trophallactic interactions could be broken down into pathways that reflect whether the nectar receiver is familiar with the flower species being collected from. Third, by providing a single, flexible framework to work within, the NBDA approach avoids the confusion that could arise if equivalent models are referred to differently depending on the nature of the network data (e.g., association rates versus records of interactions). Indeed, even in the basic NBDA model (Supplementary Equation 1), the network is ultimately broken down into a single predictor variable, $\Sigma_{j=1}^{N} a_{ij}$, such that a network framework is not necessarily required. However, for the same reasons given above, NBDA offers a practical approach for analyzing such data.

Supplementary References

1. Burnham, K. P. & Anderson, D. R. *Model selection and multimodel inference: a practical information-theoretic approach* (2nd ed.) (Springer, New York, 2002).
2. Couvillon, M. J., Schürch, R., & Ratnieks, F. L. W. Waggle dance distances as integrative indicators of seasonal foraging challenges. *PLoS ONE* **9**, e93495 (2014).
3. Garbuzov, M. & Ratnieks, F. L. W. Ivy: an underappreciated key resource to flower-visiting insects in autumn. *Insect Conserv. Divers*. **7**, 91-102 (2014).
4. Hoppitt, W. & Laland, K. N. *Social learning: an introduction to mechanisms, methods, and models* (Princeton Univ. Press, Princeton, 2013).
5. Hoppitt, W. Boogert, N. J., & Laland, K. N. Detecting social transmission in networks. *J. Theor. Biol*. **263**, 544-555 (2010).
6. Morgan, B. J. T. *Applied stochastic modelling* (2nd ed.) (Chapman & Hall/CRC Press, Boca Raton, 2009).
7. Reinhard, J., Srinivasan, M. V., & Zhang, S. Scent-triggered navigation in honeybees. *Nature* **427**, 411 (2004).
8. Johnson, D. L. Communication among honey bees with field experience. *Anim. Behav.* **15**, 487-492 (1967).
9. Grüter, C., Balbuena, M. S., & Farina, W. M. Informational conflicts created by the waggle dance. *Proc. R. Soc. B* **275**, 1321-1327 (2008).
10. Seeley, T. D. & Towne, W. F. Tactics of dance choice in honey bees: do foragers compare dances? *Behav. Ecol. Sociobiol*. **30**, 59-69 (1992).
11. Grüter, C. & Ratnieks, F. L. W. Honeybee foragers increase the use of waggle dance information when private information becomes unrewarding. *Anim. Behav*. **81**, 949-954 (2011).
