# Supplementary material for: Network-based diffusion analysis reveals context-specific dominance of dance communication in foraging honeybees
Source: Nat Commun. 2020 Jan 31;11:625. doi: 10.1038/s41467-020-14410-0 (PMC6994492; doi:10.1038/s41467-020-14410-0)
Supplement: Supplementary file 3 — Reporting Summary [file 41467_2020_14410_MOESM3_ESM.pdf]

nature research

Corresponding author(s): Matthew J. Hasenjager

Last updated by author(s): Dec 13, 2019

Reporting Summary

Nature Research wishes to improve the reproducibility of the work that we publish. This form provides structure for consistency and transparency in reporting. For further information on Nature Research policies, see Authors & Referees and the Editorial Policy Checklist .

Statistics

For all statistical analyses, confirm that the following items are present in the figure legend, table legend, main text, or Methods section.

n/a	Confirmed	
☐	☒	The exact sample size (*n*) for each experimental group/condition, given as a discrete number and unit of measurement
☐	☒	A statement on whether measurements were taken from distinct samples or whether the same sample was measured repeatedly
☐	☒	The statistical test(s) used AND whether they are one- or two-sided<br>*Only common tests should be described solely by name; describe more complex techniques in the Methods section.*
☐	☒	A description of all covariates tested
☐	☒	A description of any assumptions or corrections, such as tests of normality and adjustment for multiple comparisons
☐	☒	A full description of the statistical parameters including central tendency (e.g. means) or other basic estimates (e.g. regression coefficient) AND variation (e.g. standard deviation) or associated estimates of uncertainty (e.g. confidence intervals)
☒	☐	For null hypothesis testing, the test statistic (e.g. *F*, *t*, *r*) with confidence intervals, effect sizes, degrees of freedom and *P* value noted<br>*Give P values as exact values whenever suitable.*
☒	☐	For Bayesian analysis, information on the choice of priors and Markov chain Monte Carlo settings
☒	☐	For hierarchical and complex designs, identification of the appropriate level for tests and full reporting of outcomes
☐	☒	Estimates of effect sizes (e.g. Cohen's *d*, Pearson's *r*), indicating how they were calculated

Our web collection on statistics for biologists contains articles on many of the points above.

Software and code

Policy information about availability of computer code

Data collection	No software was used.
Data analysis	Network-based diffusion analysis was carried out using the NBDA package (ver. 0.8.3) for R available at: https://github.com/whoppitt/NBDA.

For manuscripts utilizing custom algorithms or software that are central to the research but not yet described in published literature, software must be made available to editors/reviewers. We strongly encourage code deposition in a community repository (e.g. GitHub). See the Nature Research guidelines for submitting code & software for further information.

Data

Policy information about availability of data

All manuscripts must include a data availability statement. This statement should provide the following information, where applicable:

- Accession codes, unique identifiers, or web links for publicly available datasets
- A list of figures that have associated raw data
- A description of any restrictions on data availability

Data published in this paper is provided in the Supplementary Data. The associated raw data for Supplementary Figure 1 is provided in the Source Data file.

Field-specific reporting

Please select the one below that is the best fit for your research. If you are not sure, read the appropriate sections before making your selection.

☐ Life sciences ☐ Behavioural & social sciences ☒ Ecological, evolutionary & environmental sciences

Ecological, evolutionary & environmental sciences study design

All studies must disclose on these points even when the disclosure is negative.

Study description	This study used social network analysis to identify the key social transmission pathways amongst honeybee foragers across two foraging contexts: recruitment of foragers to a never-before-visited site, and reactivation of foragers to a known site following an interruption in foraging. Three types of interaction were the focus of this study: following waggle dances, engaging in trophallactic nectar donation, and antennation. Separate social networks were constructed for each of the three interaction types, and were the key predictor variables of interest for this study. The experimental design involved training two forager groups from a single colony to artificial feeders; the experiment was repeated four times, using a different colony each time. During testing, the order that individuals arrived at each feeder was recorded, along with all social interactions that occurred within the hive. Where appropriate, we included colony ID, number of visits to an individual's familiar feeder, and foraging experience as covariates. Across the 4 experimental trials, we trained 200 individually marked foragers to our artificial feeders. During a trial, one feeder was left unfilled, while the other feeder continued to provide sucrose. The forager group that had been trained to the empty feeder, upon discovering its depletion, would then be available for recruitment to the other feeder (which they had never previously visited). In total, 56 individuals were recruited in this fashion. The order of recruitment was the response variable for the corresponding network-based diffusion analysis (NBDA), with the sample size corresponding to the number of recruited individuals. We also examined reactivation (i.e. returning to a familiar feeder following an interruption in foraging) during these same trials. Analyses of reactivation patterns were carried out separately for filled feeders and for feeders that were left unfilled during the trial. The order of reactivation was the response variable for these analyses; 91 individuals were reactivated to a familiar feeder that continued to provide sucrose, and 89 were reactivated to a familiar feeder that was empty during the trial. Some individuals were excluded as subjects in these analyses (see below), resulting in sample sizes of 67 and 70 respectively.
Research sample	The research samples were female honeybee workers (Apis mellifera) that were recruited to our artificial feeders prior to each trial. Each of the 4 experimental trials drew recruits from a separate colony, each housed in an observation hive. Our choice of subjects was motivated by our specific research questions, including the ecological relevance of the honeybee waggle dance. These colonies originated from larger source colonies on-campus maintained by a trained beekeeper. All honeybee foragers are female; although exact age could not be determined, individuals begin to forage ~2 weeks after eclosion. Each forager was given a unique enamel paint mark to identify individuals; similar marking techniques have been used for nearly a century on honeybees.
Sampling strategy	Sample size was determined by the arrival of previously marked foragers at our experimental feeders during the trials. As such, it was impossible to precisely control our sample size across conditions. Rather, we aimed to initially train comparable numbers of bees to each feeder (18-30) before carrying out the trials themselves. These numbers were selected so as to make manual collection of the network data a manageable task. We aimed to carry out the maximum number of trials we could achieve in a field season (see response to 'Timing and spatial scale' below).
Data collection	Forager arrivals at each feeder during the trials were recorded on video camera. In addition, the entrance frame of the hive where the majority of forager interactions occur was filmed during the trial. From these videos, the first author (M.J.H.) manually recorded the time of arrival for each visit to each feeder, as well as recorded all dance interactions, trophallaxes, and antennation events between individuals collecting from the filled feeder and other marked individuals within the hive.
Timing and spatial scale	The training phase for the first trial commenced on August 23rd, 2017 and the final trial was completed on October 5th, 2017. Experiments were timed to coincide with decreased natural nectar flows in order to increase forager motivation to visit the artificial feeders. A one-week gap occurred between the 2nd and 3rd trials due to unavailability of a necessary research assistant.
Data exclusions	No data were excluded from the analysis of recruitment patterns. For the reactivation analyses, it was decided a priori to exclude individuals that did not have the chance to engage in our target interaction types prior to reactivation (e.g. if they were present at the feeder when the trial began). Six additional individuals were later excluded from the Reactivation NBDA for the EMPTY feeder (see Supplementary information), as their interaction patterns were atypical relative to the rest of their cohort. Please note, however, that these latter exclusions did not qualitatively alter our findings or conclusions.
Reproducibility	The experiment was repeated for four separate colonies. The experimental protocol was successful in all cases. The number of recruits varied substantially across trials, with fewer recruits in the latter two trials relative to the first two. However, repeating the recruitment analysis using only the first two trials yielded the same findings as when all four trials were analyzed together.
Randomization	Foragers self-selected through being recruited to one of the two feeders during the training phase of each trial. Any forager that switched between feeders during training was captured and not used in the trial itself, though this event was rare. Within each trial, one feeder was left filled while the other was emptied. This was done so that foragers trained to the latter feeder, upon finding it to be empty, would be amenable to being recruited to the alternative, novel-to-them foraging site. As such, all individuals trained to the feeder left unfilled made up pool of potential recruits for each trial. All individuals trained to the filled feeders made up the pool of subjects for the Reactivation analysis presented in the main text (i.e. to the FULL feeder); likewise, all individuals trained to the unfilled feeders made up the subjects for the complementary Reactivation analysis presented in the Supporting information (i.e. to the EMPTY feeder). Which of the feeders was emptied during the trial was randomly determined, under the constraint that of the four sites used in this study, each was always used as the EMPTY site once. To control for their effects, we included the following covariates: colony ID, the number of days experience at an individual's familiar feeder (reactivation analyses), and the number of revisits to the empty feeder (recruitment analysis).
Blinding	Behavior differs dramatically between honeybees that are currently exploiting a profitable resource and those that are currently unemployed (i.e. the former produce waggle dances and make repeated, regular exits from the hive). Likewise, it is immediately obvious whether a feeder is empty or filled when recording arrivals at it. As such, it was not possible to remain blinded as to the

informational status of each forager when recording data from the videos. It was also not possible to secure assistance from someone not involved in the trials themselves to record the needed data from the videos.

Did the study involve field work? ☐ Yes ☒ No

Reporting for specific materials, systems and methods

We require information from authors about some types of materials, experimental systems and methods used in many studies. Here, indicate whether each material, system or method listed is relevant to your study. If you are not sure if a list item applies to your research, read the appropriate section before selecting a response.

Materials & experimental systems

n/a	Involved in the study
☒	☐ Antibodies
☒	☐ Eukaryotic cell lines
☒	☐ Palaeontology
☐	☒ Animals and other organisms
☒	☐ Human research participants
☒	☐ Clinical data

Methods

n/a	Involved in the study
☒	☐ ChIP-seq
☒	☐ Flow cytometry
☒	☐ MRI-based neuroimaging

Animals and other organisms

Policy information about studies involving animals; ARRIVE guidelines recommended for reporting animal research

Laboratory animals	Domestic honeybees (Apis mellifera); female workers of foraging age (> 2 weeks post-eclosion)
Wild animals	The study did not involve wild animals.
Field-collected samples	The study did not involve samples collected from the field.
Ethics oversight	The Research Ethics Committee of Royal Holloway University of London.

Note that full information on the approval of the study protocol must also be provided in the manuscript.
