# Supplementary material for: Network-based diffusion analysis reveals context-specific dominance of dance communication in foraging honeybees
Source: Nat Commun. 2020 Jan 31;11:625. doi: 10.1038/s41467-020-14410-0 (PMC6994492; doi:10.1038/s41467-020-14410-0)
Supplement: Supplementary file 4 — Description of Additional Supplementary Files [file 41467_2020_14410_MOESM4_ESM.pdf]

Description of Additional Supplementary Files

Supplementary Data 1

Description: Includes the following time-ordered interaction lists from which the networks used in the network-based diffusion analyses were constructed:

- Antennal contact_Interaction list_Duration.csv
- Antennal contact_Interaction list_Number of interactions.csv
- Antennal contact_Interaction list_First two trials_Duration.csv
- Antennal contact_Interaction list_First two trials_Number of interactions.csv
- Trophallaxis_Interaction list_Duration.csv
- Trophallaxis_Interaction list_Number of interactions.csv
- Trophallaxis_Interaction list_First two trials_Duration.csv
- Trophallaxis_Interaction list_First two trials_Number of interactions.csv
- Dance following_Interaction list_Duration.csv
- Dance following_Interaction list_Runs followed.csv
- Dance following_Interaction list_First two trials_Runs followed.csv
- Homogeneous_Interaction list_Recruitment.csv
- Homogeneous_Interaction list_First two trials_Recruitment.csv
- Homogeneous_Interaction list_Reactivation.csv

Two additional files (Number of return visits to familiar feeder_Recruitment diffusion.csv; Number of return visits to familiar feeder_Recruitment diffusion_First two trials.csv) provide mean-centred values for potential recruits of the number of visits they have made to their familiar feeder at each recruitment event. The file 'Individual-level Data.csv' provides additional information relevant for the network-based diffusion analyses for each individual forager.

Supplementary Software 1

Description: Includes R scripts to reproduce all values reported in the main text and Supplementary Information for the recruitment diffusion (Recruitment NBDA.R), reactivation diffusions (Reactivation NBDA_FULL Feeder.R; Reactivation NBDA_EMPTY Feeder.R), and for the recruitment diffusion using data from only the first two trials (Recruitment NBDA_First two trials only.R). A README file (Supplementary data README.txt) is also included to provide further information on these scripts and on the data files included in Supplementary Data 1.
